# Equine Polyclonal Antibodies Prevent Acute Chikungunya Virus Infection in Mice

**DOI:** 10.3390/v15071479

**Published:** 2023-06-29

**Authors:** Douglas Barker, Xiaobing Han, Eryu Wang, Ashley Dagley, Deborah M. Anderson, Aruni Jha, Scott C. Weaver, Justin Julander, Cory Nykiforuk, Shantha Kodihalli

**Affiliations:** 1 Emergent BioSolutions Canada Inc., Winnipeg, MB R3T 5Y3, Canada; 2 Institute for Human Infections and Immunity, Department of Microbiology and Immunology, University of Texas Medical Branch Galveston, Galveston, TX 77555, USA; 3 Institute for Antiviral Research, Utah State University, Logan, UT 84322, USA

**Keywords:** chikungunya, pharmacodynamics, mouse model

## Abstract

Chikungunya virus (CHIKV) is a mosquito-transmitted pathogen that causes chikungunya disease (CHIK); the disease is characterized by fever, muscle ache, rash, and arthralgia. This arthralgia can be debilitating and long-lasting, seriously impacting quality of life for years. Currently, there is no specific therapy available for CHIKV infection. We have developed a despeciated equine polyclonal antibody (CHIKV-EIG) treatment against CHIKV and evaluated its protective efficacy in mouse models of CHIKV infection. In immunocompromised (IFNAR^−/−^) mice infected with CHIKV, daily treatment for five consecutive days with CHIKV-EIG administered at 100 mg/kg starting on the day of infection prevented mortality, reduced viremia, and improved clinical condition as measured by body weight loss. These beneficial effects were seen even when treatment was delayed to 1 day after infection. In immunocompetent mice, CHIKV-EIG treatment reduced virus induced arthritis (including footpad swelling), arthralgia-associated cytokines, viremia, and tissue virus loads in a dose-dependent fashion. Collectively, these results suggest that CHIKV-EIG is effective at preventing CHIK and could be a viable candidate for further development as a treatment for human disease.

## 1. Introduction

Chikungunya virus (CHIKV) is a single-stranded, non-segmented, positive-sense RNA virus belonging to genus Alphavirus of the family *Togaviridae* [1,2,3]. Originally isolated in Tanzania [2,3], CHIKV is enzootic/endemic to Africa and Asia, where several outbreaks and sporadic infections occurred in the 1960s and 1970s. More recently, CHIKV infections have re-emerged on a larger scale in the Indian Ocean, South and Southeast Asia, and the Americas [2]. The range of habitat of the primary transmission vector, *Aedes aegypti*, makes large portions of the world vulnerable to the spread of CHIKV, including Western Sub-Saharan Africa, Eastern Africa, the Indian subcontinent, Southeast Asia, Northeast Australia, and the new world from Uruguay to the Southeastern United States [4]. This broad distribution has been exacerbated by mutations of CHIKV, which increase the viral infectivity of another species of mosquito, *Aedes albopictus* [5]. The infection of this vector by CHIKV is significant as *Ae. albopictus* can survive in more temperate climates and is rapidly expanding throughout the world, resulting in the expansion of areas of the world vulnerable to CHIKV infection, including most of Europe and the United States [3,6]. Thus, despite initially being considered a tropical infectious agent, CHIKV is now considered a global health challenge. Despite the global nature of the disease and its significant impact on quality of life and economic burden, no specific treatment options or vaccines are currently available. Given these limitations in prevention and control, it is highly likely that CHIKV and its insect vector will continue to spread, increasing the risk of CHIKV infection worldwide.

CHIKV infection is characterized by high fever, rash, headache, myalgia, and polyarthralgia, from which most patients fully recover once the virus has been cleared from circulation [7,8,9,10,11]. Unfortunately, acute infection can lead to chronic disease in which patients experience persistent joint and muscle pain lasting several months to years, impacting their quality of life [12,13,14,15,16].

The main emphasis for CHIKV control has been on vaccine development [17,18,19,20,21,22,23,24,25,26], which represents the best prevention strategy due to the limited antigenic diversity among CHIKV strains. Immunotherapy using polyclonal antibodies (pAbs) and monoclonal antibodies (mAbs) is a promising strategy for therapeutic and post-exposure interventions against emerging infectious diseases, which can spread rapidly in immunologically naïve populations. Antibodies have been used for passive immunization against infectious diseases for more than a century [27] and have the potential to provide immediate protection compared with vaccines, which require time to induce protective effects. Historically, passive immunotherapy with pAbs has shown prophylactic efficacy against smallpox infection and therapeutic efficacy against vaccinia infection in humans [28,29]. More recent examples of the use of passive immunotherapy with pAbs in animal models for the treatment of infectious diseases include target viruses such as Ebola [30,31,32], pandemic influenza H5N1 [33], Zika [34], vaccinia [35], SARS [36], and SARS-CoV-2 [37,38].

CHIKV replicates in peripheral tissues, resulting in high viral loads, which, in the acute phase of infection, represent a significant risk factor for the development of chronic disease [13,39]. Therefore, mAbs, which block receptor binding, prevent membrane fusion, and inhibit viral budding from infected cells, have been tested and shown to be effective when administered in the early stage of acute infection in animal models [40,41,42,43].

Despite these promising data, mAbs have several limitations, including the development of escape mutants and high production costs [44,45,46]. In contrast, the ability of pAbs to target multiple epitopes enables them to be less sensitive to viral antigenic evolution than mAbs. Additionally, pAbs exert effectiveness through diverse mechanisms of action. Polyclonal immune globulin therapeutics derived from horses present an attractive approach that can offer rapid scale-up in response to outbreaks while countering the selection of antibody escape mutants by targeting multiple vulnerable epitopes of a given pathogen. The clinical safety of equine immune globulin products is well established due to their long history in the clinic to treat several human diseases, including botulism [47,48], rabies [49], and diphtheria [50,51].

In this report, we describe the generation of an equine-derived CHIKV-specific F(ab’)_2_ antibody (CHIKV-EIG) by immunization of horses with a chimeric virus vaccine encoding CHIKV structural proteins. We further demonstrate the efficacy of CHIKV-EIG in immunocompromised and immunocompetent mouse models of CHIKV infection. In immunocompromised mice, CHIKV-EIG significantly enhanced survival and reduced viremia even when administered 24 h after infection. In immunocompetent mice, CHIKV-EIG significantly reduced footpad swelling, the viral load in serum and tissues, and the induction of cytokines associated with arthralgia. These results demonstrate that CHIKV-EIG could effectively treat acute disease and the associated deleterious effects of arthralgia in humans.

## 2. Materials and Methods

### 2.1. Materials and Ethics Statement

This research was conducted in the BSL-3 facilities at the University of Texas Medical Branch (UTMB; Galveston, TX, USA) and Utah State University (USU; Logan, UT, USA), in compliance with the Animal Welfare Act [52] and other federal statutes and regulations. All experiments involving animals adhered to the principles stated in the Guide for the Care and Use of Laboratory Animals [53] and were conducted under protocols approved by Institutional Animal Care and Use Committees.

### 2.2. Equine Immunization, Plasmapheresis, Hyperimmune Product Manufacturing

#### 2.2.1. Immunogen

An insect-specific Eilat virus (EILV)-based chimeric vaccine encoding EILV non-structural and CHIKV (99659 strain) structural proteins (EILV/CHIKV) was produced in mosquito cells as previously described [54] for use as an immunogen to hyperimmunize horses. The EILV/CHIKV chimeric virus has a particle structure identical to wild-type CHIKV and is incapable of replicating in vertebrate cells yet elicits neutralizing antibodies that protect against CHIKV in animal models [24]. Titers of virus stocks were determined by plaque assay on C7/10 mosquito cells as described below.

#### 2.2.2. Hyperimmunization

EILV/CHIKV immunizations were conducted at three-week intervals over 18 weeks, beginning with a dose of 1 × 10^5^ PFU without adjuvant. The immunogen dose was increased to 5 × 10^5^ PFU with TiterMax Gold adjuvant added at a 1:1 (*v*/*v*) ratio to obtain higher titers for the manufacturing of a hyperimmune product. Titers of the product and horse serum were determined by the plaque reduction neutralization test (PRNT) assay, as described below.

#### 2.2.3. CHIKV-EIG Manufacturing

CHIKV-EIG is a purified equine IgG product manufactured using plasma collected from horses after Day 126 of the immunization schedule by plasmapheresis. Established, validated processes were used for the manufacturing of CHIKV-EIG [55]. This lot (PD_740_POC_17_001_001) contained a total protein concentration of 49 mg/mL (>97% Fab, F(Ab’)_2_, and F(Ab’)_2_-related fragments, ≤2% monomeric IgG). Product titer was determined using a PRNT assay against CHIKV strains of Asian/American, Indian Ocean, and African lineages following previously established methods [24].

### 2.3. Assays

#### 2.3.1. Plaque Assays

EILV/CHIKV titrations were conducted using a plaque-forming assay on C7/10 mosquito cells as previously described [56]. Briefly, wells of confluent C7/10 cell monolayers in six-well plates were infected in duplicate with 200 μL of 10-fold serial dilutions of virus in DMEM + 1% FBS + 0.1% gentamycin. After one hour of adsorption, cells were overlaid with 2 mL 2% Tragacanth solution prepared in sterile water and diluted 1:1 with 2× MEM + 10% FBS + 2% tryptose phosphate broth + 0.2% gentamycin. Cells were incubated at 28 °C for 2.5 to 3 days, after which the overlay was replaced with 10% formaldehyde to fix the monolayers. Cells were then stained with crystal violet solution and plaques were counted to determine plaque-forming units (PFUs) per mL.

#### 2.3.2. Plaque Reduction Neutralization Tests

Plaque reduction neutralization tests were performed in a manner similar to those previously described [24]. The neutralization titer of equine serum samples was assessed against attenuated vaccine strain CHIKV 181/25 [57]. The neutralization titer of the CHIKV-EIG product was assessed against various strains of CHIKV (CHIKV 181/25; CHIKV 99,659 (Asian/American Lineage); Strain 37,997 (West African Lineage); Strain LR (Indian Ocean Lineage)) by PRNT assay, performed as previously described using CHIKV 181/25 as the control virus [24,58]. Briefly, plaque assays were performed on Vero cells in 12-well plates using 2-fold dilutions of sample/product starting with 1:20. Relevant virus controls and positive and negative control samples were included for each batch of samples tested on a given day. Titers were reported as a PRNT_80_ titer, the reciprocal of the highest dilution of sample inhibiting 80% of plaques.

#### 2.3.3. Infectious Cell Culture Assays

Infectious cell culture assays were performed as described previously on Vero 76 cells [59]. Serum and homogenized tissue samples were serially diluted (1:10), 100 µL of each dilution was added to Vero 76 cells in triplicate, and plates were incubated at 37 °C with 5% CO_2_ for three days and the cytopathic effect determined. Virus titers were determined by end-point titration as described previously [60] and were reported as CCID_50_ per mL (serum) or per gram (limb tissue).

#### 2.3.4. Cytokine Assays

Tissue homogenates were assayed as previously described [61] using a Mouse Cytokine Inflammation 14-Plex ELISA assay plate (Quansys Biosciences, Logan, UT, USA; catalog #110449MS). For this work, the right hindlimbs of animals were harvested at the time of sacrifice and homogenized. Cytokines assayed included GMCSF, IFNγ, IL1a, IL1b, IL2, IL3, IL4, IL5, IL6, IL10, IL12p70, IL17, MCP1, MIP1a, RANTES, and TNFα.

### 2.4. Animals

Immunocompromised IFNαβR^−/−^ mice were housed at UTMB in a facility consisting of animal rooms with separate housing for quarantine and infectious animal work. Animals were identified using ear punches and segregation into cages by study group. Homogeneity of groups by weight was the criterion used for animal randomization into treatment groups.

Immunocompetent DBA/1J mice were housed at USU in micro-isolator cages with a ventilated rack system in a facility under specific pathogen-free conditions consisting of animal rooms with separate housing for quarantine and infectious animal work. Animals were identified using ear tags.

At both UTMB and USU, food and water were provided ad libitum, and animals were randomized by weight to study groups.

### 2.5. Therapeutic Evaluation of CHIKV-EIG in Immunocompromised Mice

#### Study Design

A total of thirty (30) 7–8-week-old gender-balanced A129 IFNαβR^−/−^ mice were obtained from the colony maintained at the University of Texas Medical Branch (Galveston, TX, USA). IFNαβR^−/−^ mice are an immunocompromised strain homozygous for the type 1 interferon receptor knock-out mutation that has been used extensively to study CHIKV pathogenesis and the efficacy of anti-CHIKV vaccines and therapeutics [21,24,62,63,64,65,66,67]. Mice were infected intradermally (i.d) in the right rear footpad with 10^3^ PFU of CHIKV strain 99,659. Treatment groups were administered CHIKV-EIG intra-peritoneally (i.p.) daily for five consecutive days, beginning 5 h or 1 day after infection (post-exposure), at a dose of 100 mg/kg/day in a final volume of 100 μL. Vehicle control animals were administered 100 μL PBS i.p. daily for five consecutive days, beginning 1 day post-infection (dpi). Animals were monitored daily for clinical signs of infection, body weight loss, and survival. Severely ill animals were closely monitored, and moribund animals were euthanized according to the test facility procedures. The primary endpoint for efficacy was survival at 21 dpi. Retro-orbital blood samples were collected from surviving female animals at 1, 3, and 9 dpi and surviving male animals at 2, 4, and 9 dpi for infectious virus quantification. Terminal serum samples from all animals were also collected by cardiac puncture for infectious virus quantification and anti-CHIKV antibody titers by PRNT_80_ assay.

Viral load and antibody assessment in serum samples were performed as described above. Anti-CHIKV antibody titers in terminal serum samples from all animals surviving to the end of the study were assayed using PRNT_80_, following methods described above [24].

Group sizes were calculated to provide at least 80% power when α = 0.05 for the primary endpoints of survival at 21 dpi, assuming no control animal survival and at least 60% survival in treated animals with no correction for multiple comparisons.

### 2.6. Therapeutic Evaluation of CHIKV-EIG in Immunocompetent Mice

#### Study Design

A total of seventy-five (75), 7–8-week-old female DBA/1J mice obtained from Jackson Laboratory (Bar Harbour, ME, USA) were used. DBA/1J mice are wild-type, immunocompetent animals representing a well-characterized test system to study CHIKV pathogenesis and therapeutics [59,61,68]. The disease in this model is characterized by high viral loads, joint swelling, and muscle degeneration when infected in a footpad (either subcutaneously (s.c.) or i.d.). CHIKV-EIG was administered i.p. daily for three or five consecutive days, beginning four hours before infection (pre-exposure), at a dose of 25, 50, or 100 mg/kg/day in a final volume of 100 μL i.p. Vehicle control animals were administered 100 μL PBS i.p. daily for five consecutive days, beginning four hours before infection. An anti-inflammatory control, methotrexate (Sigma, St Louis, MO, USA), was administered at 2 mg/kg/day s.c. daily for five consecutive days, beginning one day before infection. Mice were infected with 10^5.5^ CCID_50_ CHIKV strain LR-OPY1, an Indian Ocean lineage strain isolated in 2006 from a patient on La Reunion Island, in a final volume of 100 µL administered to one footpad (s.c. or i.d.). Clinical monitoring included measurement of body weights and observations for clinical signs of infections or adverse events from 1 to 7 dpi and at 9, 11, 14, and 21 dpi. The primary efficacy endpoint was the reduction in swelling of the infected footpad. Additional endpoints included a treatment-related decrease in virus load and tissue cytokines.

Footpad swelling of the inoculated (right) foot and the contralateral (un-inoculated, left) foot was measured daily from 5 to 9 dpi with digital calipers. For each animal, footpad swelling was calculated as the percent increase or decrease in infected foot width compared to contemporaneous measurements of the contralateral foot of the same animal.

Serum was collected at 2 dpi to assess the viral load, and tissue (left and right hindlimb) samples were collected at 6 dpi to assess the viral load and tissue cytokines. Infectious virus titers in serum (n = 10) and tissue (n = 7) were determined by infectious cell culture assays as described above. Cytokines were assayed as described above.

Group sizes were calculated to provide at least 90% power when α = 0.05 for the primary endpoint of infected footpad swelling, assuming an average effect size of 29 ± 24 percent of uninfected footpad width.

### 2.7. Statistical Analyses

Survival rates in immunocompromised mice were analyzed using a two-sided Fisher’s exact test with Bonferroni–Holm adjustment. Differences in median time to death were estimated by Kaplan–Meier analysis and compared with Sidak-adjusted log-rank tests. Log-transformed serum viral load (n = 5) data were compared between groups using Dunnett’s test at each time point. Serum samples below the limit of detection (LOD) were assigned a value of 5 PFU/mL.

Footpad swelling data (n = 10), tissue cytokine data (n = 7), and log-transformed serum (n = 10) and tissue (n = 7) viral load data from immunocompetent mice were analyzed using a Wilcoxon rank-sum test. Primary comparisons (change in footpad swelling in the 100 mg/kg/day CHIKV-EIG administered for five consecutive days compared to vehicle controls at 5 and 6 dpi) were Bonferroni-adjusted; other *p* values were not adjusted for multiple comparisons. For serum viral load data, samples below LOD were assigned a value of 1.67 log_10_ CCID_50_/mL, and for tissue viral load data, samples below LOD were assigned a value of 0.67 log_10_ CCID_50_ divided by the calculated weight of tissue in the assay well to yield a value in CCID_50_/g. There was no assignment of values for tissue cytokine measurements below LOD.

Statistical analyses were conducted using either Prism 5 (GraphPad Software, Inc., Boston, MA, USA) or SAS version 9.4 (SAS Institute, Cary, NC, USA). No data points were excluded from the analysis.

## 3. Results

### 3.1. Immunization of Horses and Manufacturing of Equine CHIKV-EIG Product

**Figure 1:**
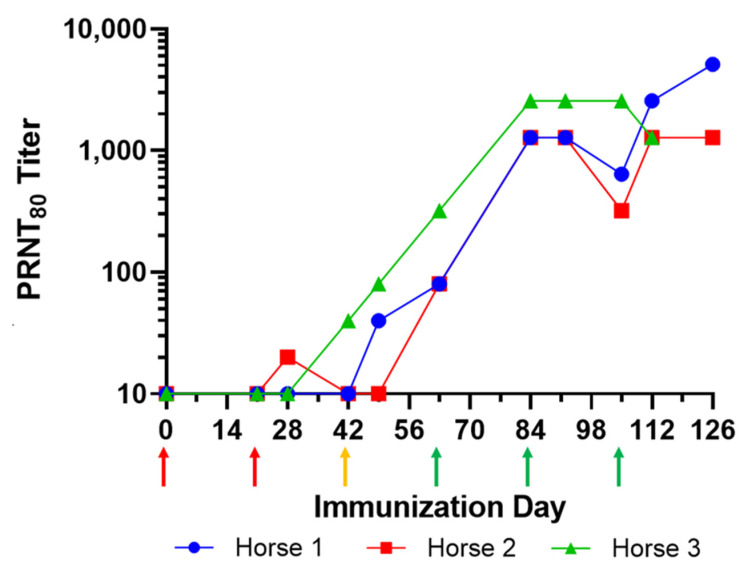
Equine response to hyperimmunization with EILV/CHIKV chimeric virus. Horses were immunized at three-week intervals, initially with 1 × 10^8^ PFU of EILV/CHIKV chimeric virus (red arrows), then 5 × 10^8^ PFU of EILV/CHIKV chimeric virus (gold arrow), and finally with 5 × 10^8^ PFU of EILV/CHIKV chimeric virus in a 1:1 ratio with TiterMax Gold adjuvant (green arrows).

Three horses were immunized with 1 × 10^8^ PFU of EILV/CHIKV chimeric virus and boosted once every 3 weeks for up to 126 days. Plasma samples were collected from each horse to evaluate the antibody response against CHIKV 181/25 using the PRNT_80_ assay. Due to a poor immune response, the immunogen dose was increased to 5 × 10^8^ PFU, beginning with the second boost on Day 42. Due to a continued limited immune response, the immunogen was supplemented with TiterMax Gold adjuvant (Titermax USA Inc., Norcross, GA, USA) at a 1:1 (*v*/*v*) ratio beginning with the third boost on Day 63. PRNT_80_ titers then increased at Day 84 to approximately 1280 and were maintained at this level to Day 126 (Figure 1). Based on the antibody titer results, plasma was collected after Day 126 from two horses (Horses 1 and 2) by plasmapheresis for manufacturing. The purified F(ab’)_2_ (CHIKV-EIG) was further evaluated by in vitro assays and mouse models of infection for efficacy.

### 3.2. In Vitro Characterization of CHIKV-EIG for Broad-Spectrum Activity

CHIKV-EIG had a high neutralizing ability against all CHIKV strains tested, including strains from all lineages (Table 1), indicating that the pAbs contained in CHIKV-EIG can neutralize diverse CHIKV strains. This is consistent with current CHIKV genotypes existing as a single serotype. In contrast, CHIKV-EIG showed varying levels of reduced cross-reactivity against related alphaviruses of the Semliki Forest antigenic complex (Table 2).

### 3.3. Protective Efficacy of CHIKV-EIG in an Immunocompromised Mouse Model

**Figure 2:**
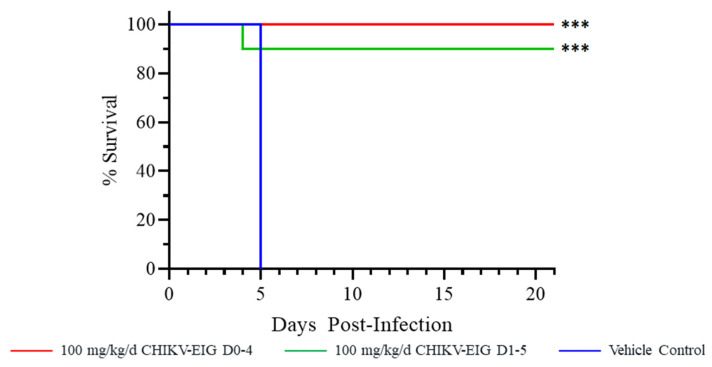
Effect of CHIKV-EIG treatment on survival of IFNαβR^−/−^ mice lethally challenged with CHIKV strain 99659. Groups of mice were administered CHIKV-EIG i.p. at 100 mg/kg daily for five consecutive days beginning 5 h or one day after infection with 10^3^ PFU of CHIKV strain 99,659 via i.d. Control animals were administered PBS via i.p. for five consecutive days beginning one day post-infection. Kaplan–Meier curves showing survival over 21 days. ***: *p* < 0.001 compared to vehicle control.

In IFNαβR^−/−^ mice, no vehicle control animals (0/10) survived to study end, with a median time to death/euthanasia of five days (Figure 2). CHIKV-EIG provided a significant (*p* < 0.001) survival benefit in both treatment groups, with 100% (10/10) of animals treated with 100 mg/kg/day CHIKV-EIG for five consecutive days beginning 5 h after infection and 90% (9/10) animals treated with 100 mg/kg/day CHIKV-EIG for five consecutive days beginning 1 dpi surviving. One animal from the group where the initiation of CHIKV-EIG treatment was delayed to 1 dpi died at 4 dpi.

**Figure 3:**
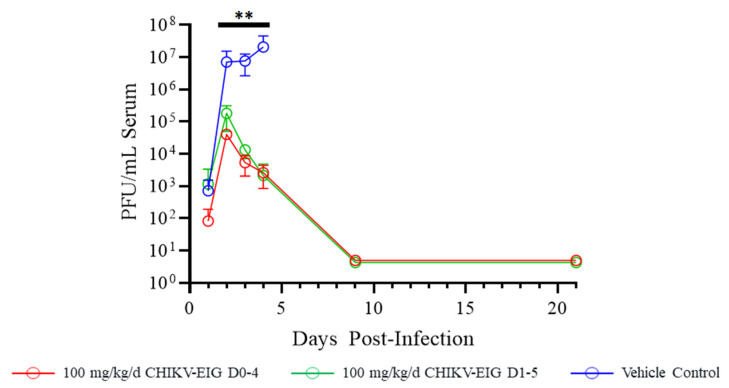
Effect of CHIKV-EIG treatment on infectious virus in serum of IFNαβR^−/−^ mice lethally challenged with CHIKV strain 99659. Groups of mice were administered CHIKV-EIG via i.p. at 100 mg/kg daily for five consecutive days beginning 5 h or one day after infection with 10^3^ PFU of CHIKV strain 99,659 via i.d. Control animals were administered PBS via i.p for five consecutive days beginning one day post-infection. Viremia data points represent the group average (± one standard deviation); infectious virus levels measured by plaque assay are presented. **: *p* < 0.01 compared to vehicle control.

Infectious virus in the serum of control animals was an average of 10^6.6^ PFU/mL at 2 dpi, increasing to an average titer of 10^7^ PFU/mL at 4 dpi. Both CHIKV-EIG treatment groups had significantly (*p* ≤ 0.007) reduced levels of virus in serum compared to controls at 2 (10^5^ PFU/mL), 3 (10^3.9^ PFU/mL), and 4 dpi (10^3.1^ PFU/mL). Surviving animals had no detectable viremia at either 9 or 21 dpi (Figure 3).

Despite the reduction in viremia, CHIKV-EIG-treated animals surviving to the end of the study at 21 dpi showed an anti-CHIKV antibody response, with neutralizing titers ranging from 40 to 320 by PRNT_80_ (Table S1), suggesting a robust immune response. Given the anticipated short half-life of CHIKV-EIG in rodents [38] due to its size, it is unlikely that this anti-CHIKV activity was due to residual CHIKV-EIG and it can be attributed to a primary immune response to infection.

**Figure 4:**
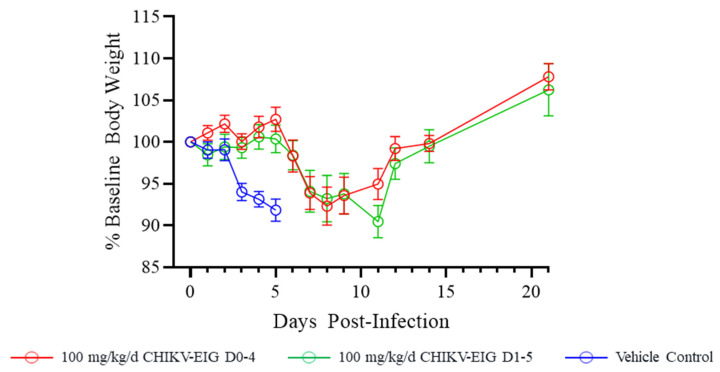
Effect of CHIKV-EIG treatment on body weight changes of IFNαβR^−/−^ mice lethally challenged with CHIKV strain 99,659. Groups of mice were administered CHIKV-EIG via i.p. at 100 mg/kg daily for five consecutive days beginning 5 h or one day after infection with 10^3^ PFU of CHIKV strain 99,659 via i.d. Control animals were administered PBS via i.p. for five consecutive days beginning one day post-infection. Data points represent the average (±one standard error of the mean) percentage of baseline body weight for each day for each study group.

Sham-treated control animals lost weight daily from 1 dpi until the animals succumbed at 5 dpi. Peak body weight loss was 8.17% at 5 dpi (average weight 91.83% of baseline body weight). The time to peak body weight loss was significantly delayed by CHIKV-EIG treatment compared to controls (*p* ≤ 0.0057), with CHIKV-EIG-treated animals beginning to lose weight at 6 dpi, with peak weight loss at either 8 or 11 dpi, respectively, in animals treated on the same day of infection or delayed by 1 day after infection This suggests a treatment-related delay in morbidity onset (Table 3). This weight loss in CHIKV-EIG treated animals was transient, with average body weights returning to baseline by 12 dpi (Figure 4). Weight loss as a proportion of baseline weight was consistently greater for male animals compared to females (Table S2).

### 3.4. Protection against Effects of CHIKV in an Immunocompetent Mouse Model

**Figure 5:**
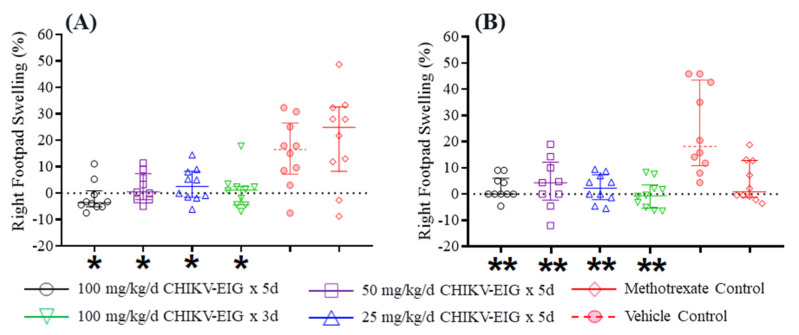
Median footpad swelling in DBA/1J mice infected subcutaneously with CHIKV strain LR-OPY1 is significantly reduced by treatment with CHIKV-EIG at (**A**) 5 dpi and (**B**) 6 dpi. Groups of mice administered various dose levels of CHIKV-EIG via i.p. route (100, 50, 25 mg/kg) daily for either five days, or (100 mg/kg) for three days. All CHIKV-EIG treatments were initiated 4 h prior to infection with 10^5.5^ CCID_50_ CHIKV strain LR-OPY1. Control animals were administered via i.p. for five days beginning 4 h prior to infection, or administered methotrexate s.c. at 2 mg/kg/day for five days beginning one day prior to infection. Data points represent group median increase and interquartile range in footpad thickness of the infected (right hind) footpad compared to the uninfected (left hind) footpad. *: *p* ≤ 0.05 compared to vehicle control. **: *p* < 0.01 compared to vehicle control.

The protective effect of CHIKV-EIG was also evaluated in immunocompetent DBA/1J mice. All sham-treated control animals exhibited swelling of the infected footpad, confirming the ability of the virus to elicit the expected clinical effects. Footpad swelling of the infected foot was significant in control animals, with peak swelling of over 50% compared to the uninfected foot at 7 dpi. The footpad swelling in methotrexate-treated animals was reduced compared to vehicle controls at 6 dpi but was increased at 8 and 9 dpi (Table S3). The peak level of swelling in the infected foot in these animals was similar to that observed in the control group, consistent with previous studies in this model [61,68]. In contrast, all groups treated with CHIKV-EIG had little or no footpad swelling on any of the days tested; this reduced footpad swelling was significant compared to controls at 5 (*p* ≤ 0.020) and 6 dpi (*p* ≤ 0.008) (Figure 5, Table S4). Treatment with 100 mg/kg/day CHIKV-EIG for either three or five consecutive days induced the same level of reduction in the footpad swelling.

**Figure 6:**
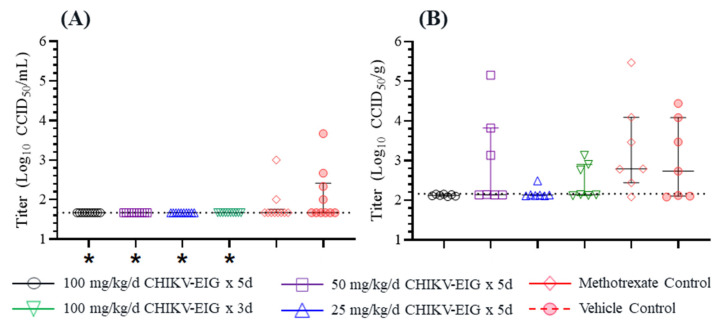
Infectious CHIKV strain LR-OPY1 titers are reduced in (**A**) at 2 dpi in serum and (**B**) at 6 dpi in infected hindlimb tissues of DBA/1J mice by treatment with CHIKV-EIG. Groups of mice administered various dose levels of CHIKV-EIG via i.p. route (100, 50, 25 mg/kg) daily for five days or (100 mg/kg) for three days. All CHIKV-EIG treatments were initiated 4 h prior to infection with 10^5.5^ CCID_50_ CHIKV strain LR-OPY1. Control animals were administered via i.p. for five days beginning 4 h prior to infection, or administered methotrexate s.c. at 2 mg/kg/day for five days beginning one day prior to infection. Individual data points represent single animals; horizontal lines represent group median values and interquartile ranges. *: *p* < 0.05 compared to vehicle control.

Infectious virus was undetectable in serum from CHIKV-EIG-treated animals at 2 dpi, the only timepoint where serum was collected. The levels were significantly (*p* = 0.035) lower for all CHIKV-EIG treatment groups regardless of the dose or duration of treatment, despite most control animals lacking detectable levels of viremia (Figure 6A). As with controls, most of the methotrexate-treated animals lacked detectable virus in serum. Infectious virus was also quantified in tissue from the inoculated (right hind) limb at 6 dpi (Figure 6B). The mean viral load in right hindlimb tissue was lower in all CHIKV-EIG treatment groups compared to control and methotrexate-treated groups. The viral load also was reduced significantly for the groups treated with 100 mg/kg/day CHIKV-EIG (five-day treatment regimen) and 25 mg/kg/day CHIKV-EIG groups (*p* = 0.030 and *p* = 0.041, respectively) compared to the group treated with methotrexate. There were no significant differences in tissue virus titers in control and CHIKV-EIG-treated groups at any dose level tested.

**Figure 7:**
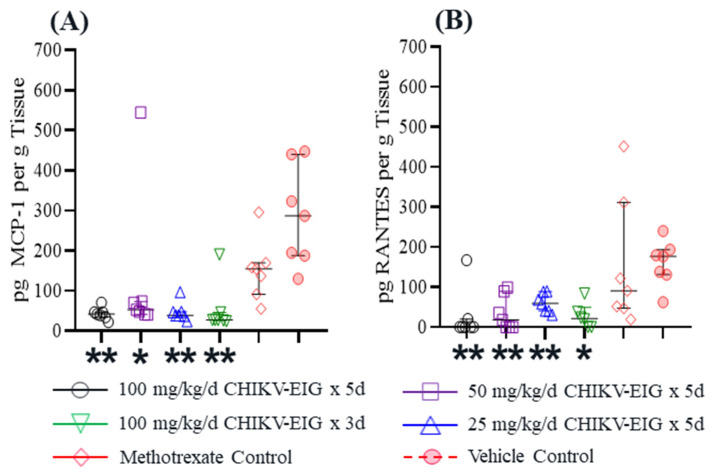
Reduction in (**A**) MCP1 and (**B**) RANTES expression by treatment with CHIKV-EIG in infected hindlimb tissue of DBA/1J mice at 6 dpi. The cytokines (**A**) MCP1 and (**B**) RANTES were quantified in infected hindlimb tissue at 6 dpi (n = 7 per group). Groups of mice administered various dose levels of CHIKV-EIG via i.p. route (100, 50, 25 mg/kg) daily for five days or (100 mg/kg) for three days. All CHIKV-EIG treatments were initiated 4 h prior to infection with 10^5.5^ CCID_50_ CHIKV strain LR-OPY1. Control animals were administered via i.p. for five days beginning 4 h prior to infection, or administered methotrexate s.c. at 2 mg/kg/day for five days beginning one day prior to infection. Symbols represent individual animals. Horizontal lines represent group median values and interquartile ranges. *: *p* < 0.05 compared to vehicle control; **: *p* < 0.01 compared to vehicle control.

CHIKV-EIG treatment significantly reduced the levels of MCP1 (*p* ≤ 0.03) and RANTES (*p* ≤ 0.041) compared to vehicle controls by at least 5-fold and 2.9-fold, respectively (Figure 7, Table S5). MCP1 was significantly (*p* ≤ 0.021) reduced in all CHIKV-EIG treatment groups compared to the methotrexate-treated group, except in the 50 mg/kg/day dose group (*p* = 0.055). Significant reductions in RANTES levels in the CHIKV-EIG group (50 mg/kg/day) compared to the methotrexate-treated group were also observed (*p* ≤ 0.04).

## 4. Discussion

CHIKV-EIG is an investigational product derived from the plasma of horses hyperimmunized with a CHIKV/EILV chimeric virus. This report demonstrates the efficacy of CHIKV-EIG as a treatment for acute CHIKV infection in an immunocompetent mouse model in a pre-exposure prophylaxis setting and in an immunocompromised mouse model in a post-exposure prophylaxis setting.

CHIKV-EIG treatment significantly enhanced survival (Figure 2) and reduced the virus load in the serum (Figure 3) of IFNαβR^−/−^ mice infected with CHIKV strain 99,659. The onset of body weight loss was delayed until after CHIKV-EIG treatment ceased (Figure 4, Table S2). The rate and magnitude of weight loss were comparable to sham-treated controls despite the reduced viral load, highlighting the sensitivity of this model. The use of CHIKV-EIG did not impede the development of an anti-CHIKV immune response in surviving animals, with anti-CHIKV antibody titers of 40 to 320 PRNT_80_ observed at 21 dpi (Table S1).

The survival benefit offered by CHIKV-EIG in this model is consistent with the findings for a human hyperimmune product administered as a single dose at the same time as infection [66] and a two monoclonal antibody cocktail administered as a single dose up to 24 h after infection [64]. Interestingly, one of the monoclonal antibodies in this study (CHK152 N297Q) had significantly reduced efficacy when the Fc effector function was eliminated via an N297Q mutation in comparison to CHIK152, indicating a contribution from the Fc effector function for efficacy. This was apparent only at the lower dose level of CHK152 N297Q. Although we have not compared the despeciated CHIKV-EIG equine product to a full-length equine IgG product in our mouse model studies, full protection with the CHIKV-EIG that lacks the Fc fragment has been demonstrated at a high dose level. Further studies are needed to evaluate the Fc effector function requirement for protection at a lower dose level of CHIKV-EIG.

The humoral response, especially neutralizing antibodies, is crucial in controlling and eliminating CHIKV infection and persistence [15,69] and preventing disease in naturally exposed people [70]. The polyclonal CHIKV-EIG has a high titer and cross-reactivity against Asian, Indian Ocean, and West African lineages of CHIKV strains in vitro (Table 1) and was effective against the Asian lineage CHIKV-99659 virus in immunocompromised animals (Figure 2, Figure 3 and Figure 4) and against the Indian Ocean lineage CHIKV-LR-OPY1 virus in immunocompetent animals (Figure 5, Figure 6 and Figure 7). Compared to mAb therapy, for which escape mutants have been observed [71,72,73], this cross-lineage reactivity highlights the advantage of CHIKV-EIG as a cross-protective pAb product targeting multiple epitopes.

Immunocompromised IFNαβR^−/−^ mice lack receptors for interferon–α/β, making them highly vulnerable to fatal infection with CHIKV. Therefore, these mice are useful tools to study the role of the type I IFN system in CHIKV pathogenesis and to test the efficacy of anti-CHIKV Abs or the safety and effectiveness of CHIKV vaccines [17,20,64,67]. Unfortunately, the short time to death after infection observed in this model limits its usefulness in studying the pathogenesis of CHIK or protection by antivirals [74]. Therefore, the efficacy of CHIKV-EIG was also evaluated in a non-lethal immunocompetent mouse model of CHIKV infection [15,75] to assess the effects of treatment on acute joint inflammation. During the acute infection of immunocompetent animals, sham-treated controls showed substantial swelling in the inoculated footpad compared to the contralateral (uninoculated) footpad (Figure 5, Tables S3 and S4). Unlike treatment with methotrexate, the reduction in swelling in the CHIKV-EIG treatment group was maintained after the cessation of treatment. This was likely due to the reduction in infectious CHIKV in serum at 2 dpi after infection and hindlimb tissue at 6 dpi in CHIKV-EIG treated animals compared to controls and methotrexate-treated animals (Figure 6).

These results indicate that CHIKV-EIG is superior in the immunocompetent mouse model compared to methotrexate, an anti-inflammatory agent used to treat chronic CHIK in humans [76]. This may be due to CHIKV-EIG neutralizing the underlying CHIKV infection, whereas methotrexate acts only to suppress inflammation in infected mice. The CHIKV-EIG treatment also significantly reduced viremia, indicating that rapid viral neutralization by CHIKV-EIG in circulation could reduce the dissemination of the virus to sites distal from the initial infection. Reducing the viral load during the acute phase by CHIKV-EIG treatment may limit joint disease and mitigate the development of chronic CHIK [14,77,78].

A number of cytokines and chemokines involved in the induction or regulation of inflammatory responses were shown to be associated with the severe and persistent symptoms of CHIKV infection [79,80]. Our results showed that the levels of the arthralgia-associated cytokines Monocyte Chemoattractant Protein-1 (MCP-1/CCL2) and Regulated on Activation, Normal T Cell Expressed and Secreted (RANTES) were significantly reduced by CHIKV-EIG in hindlimb tissue at 6 dpi (Figure 7, Table S5). MCP-1/CCL2 is one of the key chemokines regulating the migration and infiltration of monocytes/macrophages [81], which are known cellular reservoirs of CHIKV in the later stages of infection and play an important role in prolonged inflammation [80]. Various studies implicate RANTES as a mediator in chronic inflammation [82,83] and as a player in rheumatoid arthritis pathogenesis [84,85,86,87]. Our data from mice suggest that CHIKV-EIG treatment is capable of not only neutralizing CHIKV infection but also reducing acute CHIK pathogenesis.

When viewed together, the data presented here point towards CHIKV-EIG being a potentially useful treatment for acute CHIKV infection in humans even after the onset of detectable viremia. Antibody therapies to treat infectious diseases are recognized as most effective when delivered prophylactically or early in the course of disease [88,89], and CHIKV-EIG represents an attractive strategy to control acute infection, minimize viral persistence, and prevent long-term joint disease. Using a polyclonal product such as CHIKV-EIG may provide advantages in terms of preventing resistance to viral escape, and the ability of CHIKV-EIG to cross-neutralize CHIKV strains representative of the West African, Asian, and East/Central/South African/Indian Ocean lineages highlights the potential of this product to be used broadly against CHIKV infections worldwide.

The current work focused on the effects of CHIKV-EIG in acute CHIKV infection models using immunocompromised and immunocompetent mice. The major limitation of this study was that the disease models used did not mimic the human clinical setting. CHIKV causes chronic disease in humans, and the rapid time course of the acute disease models limits the time of intervention compared to humans. Additional studies are required in relevant animal models to assess the efficacy of CHIKV-EIG in preventing or reducing the symptoms of chronic disease.

In vitro studies have shown replication of CHIKV in human placental cells and a reduction in CHIKV replication by cross-reactive antibodies [90]. This finding is significant in addressing congenital infections with CHIKV during pregnancy using appropriate therapeutics. Further investigation is needed to assess CHIKV-EIG to prevent vertical transmission of the disease. Ultimately, human trials will be required to translate the work presented here to the clinic.

In conclusion, these data demonstrate the effectiveness of CHIVK-EIG in treating an acute CHIKV infection, with significantly enhanced survival and reduced body weight loss in immunocompromised mice, reduced infected footpad swelling in immunocompetent mice, and reduced serum and tissue viral loads in both mouse models.

## Figures and Tables

**Table 1:** In vitro neutralizing titers of CHIKV-EIG for CHIKV strains representing various lineages.

Strain	Lineage	Titer (PRNT_80_)
CHIKV 181/25	-	5120
99,659	Asian	5120
LR	Indian Ocean	2560
37,997	West African	5120

**Table 2:** In vitro neutralizing titers of CHIKV-EIG for viruses of the Semliki Forest Virus antigenic complex.

Virus	Strain	Titer (PRNT_80_)
O’Nyong-Nyong	SG650	640
Semliki Forest Virus	Kumba	40
Mayaro Virus	FMD3212	<20
	TRVL15537	20
	BeAR505411	<20
	INHRR11a-10	20
Ross River Virus	T48	<20

**Table 3:** Effect of CHIKV-EIG treatment on time to peak percent body weight loss in IFNαβR^−/−^ mice compared to vehicle controls.

Treatment Group	Mean Peak Percent Weight Loss (95% CI)	Median Time to Peak Weight Loss in Days (95% CI)	Sidak-Adjusted Log-Rank Test vs. Vehicle Control
Vehicle Control	8.17 (5.56, 10.78)	5.00 (3.00, 5.00)	n/a
CHIKV-EIG 100 mg/kg/day (qd X5, i.p. beginning 5 h post-infection)	7.70 (3.19, 12.20)	8.00 (7.00, 9.00)	0.0057 *
CHIKV-EIG 100 mg/kg/day (qd X5, i.p. beginning day 1 post-infection)	9.53 (5.71, 13.34)	11.0 (4.00, 11.0)	<0.001 *

* Denotes significant *p* value (≤0.05). *p* values are not adjusted for multiple comparisons.

## Data Availability

All the relevant data are included within the manuscript and its Supplementary Material files.

## Supplementary Materials

The following supporting information can be downloaded at: https://www.mdpi.com/article/10.3390/v15071479/s1, Table S1: Average number of plaques per duplicate well observed from serum samples collected from immunocompromised IFNαβR^−/−^ mice surviving to 21 days post CHIKV infection and calculated PRNT80 titer; Table S2: Mean percentage change in body weight from baseline in immunocompromised IFNαβR^−/−^ mice by study day, treatment group, and gender; Table S3: Summary of percent change in right footpad swelling (from left footpad) at 5 to 9 dpi in CHIKV-infected immunocompetent DBA/1J mice by treatment group; Table S4: Analysis of percent change in right footpad swelling (from left footpad) at 5 and 6 dpi in CHIKV-EIG treated immunocompetent DBA/1J mice; Table S5: Summary statistics of cytokine levels at 6 dpi in right hindlimb tissue, cytokine levels in immunocompetent DBA/1J mice, and comparison between CHIKV-EIG treatment groups and vehicle and methotrexate (anti-inflammatory) controls.
