# Supplementary material for: Equine Polyclonal Antibodies Prevent Acute Chikungunya Virus Infection in Mice
Source: Viruses. 2023 Jun 29;15(7):1479. doi: 10.3390/v15071479 (PMC10384969; doi:10.3390/v15071479)
Supplement: Supplementary file 1 [file viruses-15-01479-s001.zip › viruses-2414515-supplementary.pdf]

**Table S1.** Average number of plaques per duplicate well observed from serum samples collected from immunocompromised IFNαβR$^{-/-}$ mice surviving to 21 days post CHIKV infection and calculated $PRNT_{80}$ titer.

| Group | Animal Cage # [a] | Animal ID[b] | Dilution 1:20 | 1:40 | 1:80 | 1:160 | 1:320 | 1:640 | $PRNT_{80}$[c] |
|---|---|---|---|---|---|---|---|---|---|
| 2 [d] | 2M | 1L | 0 | 0 | 4 | 3 | 4 | 4 | 40 |
| | 2M | 1R | 0 | 0 | 1 | 3 | 3 | 6 | 160 |
| | 2M | 2L | 0 | 0 | 2 | 2 | 2 | 6 | 320 |
| | 2M | 1L2R | 0 | 0 | 0 | 1 | 2 | 8 | 320 |
| | 2M | N | 0 | 0 | 1 | 1 | 4 | 5 | 160 |
| | 2F | 1L | 2 | 1 | 3 | 4 | 5 | 5 | 80 |
| | 2F | 1R | 0 | 2 | 4 | 4 | 9 | 5 | 40 |
| | 2F | 2L | 0 | 0 | 4 | 5 | 3 | 4 | 40 |
| | 2F | 1L2R | 0 | 1 | 3 | 5 | 2 | 4 | 80 |
| | 2F | N | 0 | 0 | 2 | 4 | 5 | 7 | 40 |
| 3 [e] | 3M | 1R | 0 | 0 | 1 | 2 | 5 | 6 | 160 |
| | 3M | 2L | 0 | 0 | 1 | 4 | 3 | 5 | 80 |
| | 3M | 1L2R | 0 | 0 | 1 | 1 | 5 | 7 | 160 |
| | 3M | N | 0 | 1 | 1 | 1 | 3 | 3 | 320 |
| | 3F | 2L2R | 0 | 0 | 0 | 0 | 3 | 7 | 320 |
| | 3F | 1R | 1 | 2 | 2 | 2 | 4 | 7 | 160 |
| | 3F | 2L | 0 | 2 | 3 | 6 | 9 | 8 | 80 |
| | 3F | 1L1R | 0 | 1 | 1 | 1 | 6 | 5 | 160 |
| | 3F | N | 0 | 1 | 2 | 3 | 11 | 8 | 160 |
| Positive Mouse Control | | | 0 | 1 | 2 | 4 | 3 | 7 | 80 |
| Negative Mouse Control | | | 7 | 11 | 13 | 13 | -- | -- | <20 |

a All animals in cages with the suffix "M" were males, all animals in cages with the suffix "F" were females. b 1L = one left ear punch; 1R = one right ear punch; 2L = two left ear punches; 1L2R = one left ear punch and two right ear punches; N = no ear punches. c Defined as the reciprocal of the highest dilution where the average number of plaques per well did not exceed the calculated cut-off value of three plaques per well. d 100 mg/kg CHIKV-EIG qd X 5, i.p., beginning 5 ± 1 h after infection. e 100 mg/kg CHIKV-EIG qd X 5, i.p., beginning 22 ± 2 h after infection.

**Table S2.** Mean percent change in body weight from baseline in immunocompromised IFNαβR$^{-/-}$ mice by study day, treatment group and gender.

| Study Day | Treatment Group | Gender | Mean (95% Confidence Interval) |
|---|---|---|---|
| 1 | 1 [a] | Female Only | 1.83 (1.25, 2.41) |
| | | Male Only | −3.76 (−5.37, −2.16) |
| | | Female + Male | −0.97 (−2.88, 0.95) |
| | 2 [b] | Female Only | 1.54 (−3.15, 6.23) |
| | | Male Only | 0.65 (−1.49, 2.79) |
| | | Female + Male | 1.10 (−0.64, 2.83) |
| | 3 [c] | Female Only | −0.25 (−6.58, 6.08) |
| | | Male Only | −2.8 (−6.8, 1.2) |
| | | Female + Male | −1.52 (−4.15, 1.11) |
| 2 | 1 | Female Only | 2.53 (−0.17, 5.23) |
| | | Male Only | −4.41 (−6.63, −2.19) |

| | | | |
|---|---|---|---|
| | | Female + Male | −0.94 (−3.49, 1.61) |
| | 2 | Female Only | 2.27 (−0.63, 5.18) |
| | | Male Only | 2.03 (−3.37, 7.43) |
| | | Female + Male | 2.15 (0.11, 4.19) |
| | 3 | Female Only | −1.07 (−8.83, 6.7) |
| | | Male Only | −0.09 (−4.35, 4.17) |
| | | Female + Male | −0.58 (−3.54, 2.39) |
| 3 | 1 | Female Only | −3.28 (−6.11, −0.45) |
| | | Male Only | −8.68 (−9.89, −7.48) |
| | | Female + Male | −5.98 (−8.02, −3.94) |
| | 2 | Female Only | 0.03 (−3.11, 3.17) |
| | | Male Only | 0.03 (−4.4, 4.46) |
| | | Female + Male | 0.03 (−1.77, 1.84) |
| | 3 | Female Only | −0.69 (−7.29, 5.9) |
| | | Male Only | −0.7 (−4.2, 2.8) |
| | | Female + Male | −0.69 (−3.18, 1.79) |
| 4 | 1 | Female Only | −4.49 (−7.03, −1.94) |
| | | Male Only | −9.24 (−10.31, −8.17) |
| | | Female + Male | −6.86 (−8.67, −5.06) |
| | 2 | Female Only | 1.55 (−2.53, 5.62) |
| | | Male Only | 2.05 (−4.39, 8.5) |
| | | Female + Male | 1.80 (−0.74, 4.34) |
| | 3 | Female Only | 2.12 (−5.49, 9.72) |
| | | Male Only | −0.91 (−3.96, 2.13) |
| | | Female + Male | 0.60 (−2.30, 3.50) |
| 5 | 1 | Female Only | −5.29 (−10.63, 0.05) |
| | | Male Only | −11.05 (−11.91, −10.2) |
| | | Female + Male | −8.17 (−10.78, −5.56) |
| | 2 | Female Only | 2.58 (−2.23, 7.39) |
| | | Male Only | 2.86 (−4.15, 9.86) |
| | | Female + Male | 2.72 (−0.11, 5.55) |
| | 3 | Female Only | 2.38 (−4.15, 8.91) |
| | | Male Only | −2.14 (−8.14, 3.85) |
| | | Female + Male | 0.37 (−2.89, 3.63) |
| 6 | 2 | Female Only | 0.36 (−3.54, 4.26) |
| | | Male Only | −3.73 (−13.64, 6.18) |
| | | Female + Male | −1.69 (−5.47, 2.10) |
| | 3 | Female Only | 1.55 (−2.99, 6.1) |
| | | Male Only | −5.49 (−12.56, 1.58) |
| | | Female + Male | −1.58 (−5.03, 1.87) |
| 7 | 2 | Female Only | −2.52 (−9.29, 4.24) |
| | | Male Only | −9.7 (−16.16, −3.24) |
| | | Female + Male | −6.11 (−10.01, −2.21) |
| | 3 | Female Only | −3.8 (−13.34, 5.75) |
| | | Male Only | −8.53 (−20.41, 3.34) |
| | | Female + Male | −5.90 (−10.82, −0.98) |
| 8 | 2 | Female Only | −3.26 (−10.85, 4.32) |
| | | Male Only | −12.13 (−19.2, −5.06) |
| | | Female + Male | −7.70 (−12.20, −3.19) |
| | 3 | Female | −3.31 (−11.37, 4.75) |
| | | Male | −11.13 (−25.75, 3.49) |

| | | | |
|---|---|---|---|
| | | Female + Male | −6.78 (−12.24, −1.33) |
| 9 | 2 | Female Only | −2.97 (−9.39, 3.46) |
| | | Male Only | −9.84 (−18.92, −0.76) |
| | | Female + Male | −6.40 (−10.73, −2.08) |
| | 3 | Female Only | −3.34 (−9.78, 3.11) |
| | | Male Only | −9.8 (−23.49, 3.89) |
| | | Female + Male | −6.21 (−10.96, −1.46) |
| 11 | 2 | Female Only | −1.9 (−6.34, 2.53) |
| | | Male Only | −8.17 (−16.1, −0.25) |
| | | Female + Male | −5.04 (−8.69, −1.38) |
| | 3 | Female Only | −11.75 (−17.76, −5.74) |
| | | Male Only | −6.74 (−16.97, 3.48) |
| | | Female + Male | −9.53 (−13.34, −5.71) |
| 12 | 2 | Female Only | 0.66 (−2.11, 3.43) |
| | | Male Only | −2.22 (−9.7, 5.27) |
| | | Female + Male | −0.78 (−3.60, 2.04) |
| | 3 | Female Only | −0.58 (−7.22, 6.07) |
| | | Male Only | −5.08 (−14.0, 3.85) |
| | | Female + Male | −2.57 (−6.26, 1.11) |
| 14 | 2 | Female Only | 0.12 (−3.46, 3.71) |
| | | Male Only | −0.48 (−4.72, 3.75) |
| | | Female + Male | −0.18 (−2.04, 1.68) |
| | 3 | Female Only | 1.73 (−6.33, 9.79) |
| | | Male Only | −3.31 (−10.34, 3.72) |
| | | Female + Male | −0.51 (−4.40, 3.38) |
| 21 | 2 | Female Only | 6.64 (3.68, 9.6) |
| | | Male Only | 9.0 (0.47, 17.53) |
| | | Female + Male | 7.82 (4.72, 10.92) |
| | 3 | Female Only | 6.28 (−8.32, 20.88) |
| | | Male Only | 6.18 (−5.16, 17.52) |
| | | Female + Male | 6.24 (0.10, 12.37) |

a 100 µL PBS (vehicle) qd X 5, i.p., beginning 22 ± 2 h after infection. b 100 mg/kg CHIKV-EIG qd X 5, i.p., beginning 5 ± 1 h after infection. c 100 mg/kg CHIKV-EIG qd X 5, i.p., beginning 22 ± 2 h after infection.

**Table S3.** Summary of percent change in right footpad swelling (from left footpad) at 5 dpi to 9 dpi in CHIKV infected immunocompetent DBA/1J mice by treatment group.

| Day | Treatment Group | n | % Change in Right Footpad Swelling (From Left Footpad) Mean (SD) | Median (25th, 75th Percentile) | Min, Max |
|---|---|---|---|---|---|
| 5 | 1 [a] | 10 | −1.635 (5.648) | −3.56 (−5.07, −0.48) | −7.45, 11.11 |
| | 3 [b] | 9 | 2.245 (5.545) | 0.48 (−2.39, 5.77) | −4.91, 11.43 |
| | 5 [c] | 10 | 3.193 (6.249) | 2.56 (−1.37, 8.08) | −6.05, 14.50 |
| | 7 [d] | 10 | 1.078 (6.846) | 1.18 (−4.37, 2.38) | −6.87, 17.81 |
| | 9 [e] | 10 | 20.588 (17.416) | 24.89 (11.90, 32.37) | −8.68, 48.69 |
| | 11[f] | 10 | 15.304 (12.433) | 16.48 (8.60, 25.12) | −7.49, 32.39 |
| 6 | 1 | 10 | 2.298 (4.435) | 0.00 (0.00, 5.00) | −4.55, 9.09 |
| | 3 | 9 | 3.989 (9.552) | 4.35 (0.00, 10.00) | −12.00, 19.05 |
| | 5 | 10 | 2.304 (5.344) | 2.27 (−1.32, 7.01) | −5.44, 9.52 |
| | 7 | 10 | −0.238 (5.285) | −0.66 (−5.06, 2.13) | −6.41, 8.33 |

| | | | | | |
|---|---|---|---|---|---|
| | 9 | 10 | 4.654 (7.788) | 0.86 (−0.90, 12.78) | −3.56, 18.78 |
| | 11 | 10 | 24.429 (16.292) | 18.17 (11.81, 42.68) | 4.44, 45.89 |
| 7 | 1 | 3 | 1.329 (4.916) | 4.17 (−4.35, 4.17) | −4.35, 4.17 |
| | 3 | 2 | −4.348 (6.149) | −4.35 (−8.70, 0.00) | −8.70, 0.00 |
| | 5 | 3 | 3.235 (6.913) | 4.35 (−4.17, 9.52) | −4.17, 9.52 |
| | 7 | 3 | 0.000 (0.000) | 0.00 (0.00, 0.00) | 0.00, 0.00 |
| | 9 | 3 | 19.769 (13.769) | 22.73 (4.76, 31.82) | 4.76, 31.82 |
| | 11 | 3 | 50.922 (10.462) | 54.55 (39.13, 59.09) | 39.13, 59.09 |
| 8 | 1 | 3 | 1.515 (2.624) | 0.00 (0.00, 4.55) | 0.00, 4.55 |
| | 3 | 2 | −2.165 (9.795) | −2.16 (−9.09, 4.76) | −9.09, 4.76 |
| | 5 | 3 | 9.596 (12.476) | 14.29 (−4.55, 19.05) | −4.55, 19.05 |
| | 7 | 3 | 4.414 (4.349) | 4.55 (0.00, 8.70) | 0.00, 8.70 |
| | 9 | 3 | 50.412 (11.025) | 47.83 (40.91, 62.50) | 40.91, 62.50 |
| | 11 | 3 | 21.465 (12.548) | 22.73 (8.33, 33.33) | 8.33, 33.33 |
| 9 | 1 | 3 | 0.000 (0.000) | 0.00 (0.00, 0.00) | 0.00, 0.00 |
| | 3 | 2 | 2.489 (9.948) | 2.49 (−4.55, 9.52) | −4.55, 9.52 |
| | 5 | 3 | −4.282 (7.645) | −8.70 (−8.70, 4.55) | −8.70, 4.55 |
| | 7 | 3 | 1.515 (2.624) | 0.00 (0.00, 4.55) | 0.00, 4.55 |
| | 9 | 3 | 34.327 (17.264) | 26.09 (22.73, 54.17) | 22.73, 54.17 |
| | 11 | 3 | 5.863 (6.621) | 4.55 (0.00, 13.04) | 0.00, 13.04 |

p.i. = post-infection; SD = standard deviation. a 100 mg/kg/d CHIKV-EIG qd X 5, i.p., beginning 4 h before infection. b 50 mg/kg/d CHIKV-EIG qd X 5, i.p., beginning 4 h before infection. c 25 mg/kg/d CHIKV-EIG qd X 5, i.p., beginning 4 h before infection. d 100 mg/kg/d CHIKV-EIG qd X 3, i.p., beginning 4 h before infection. e 2 mg/kg/d methotrexate qd X 5, s.c., beginning 1 day before infection. f 100 µL PBS (vehicle) qd X 5, i.p., beginning 4 h before infection.

**Table S4.** Analysis of percent change in right footpad swelling (from left footpad) at 5 dpi and 6 dpi in CHIKV-EIG treated immunocompetent DBA/1J mice.

| Day | Treatment Group | Wilcoxon Rank-Sum Test *p* Value Versus Vehicle Control | Wilcoxon Rank-Sum Test *p* Value Versus Methotrexate Control |
|---|---|---|---|
| 5 | 1 [a] | 0.006 * | 0.015 * |
| | 3 [b] | 0.025 * | 0.020 * |
| | 5 [c] | 0.031 * | 0.017 * |
| | 7 [d] | 0.021 * | 0.009 * |
| 6 | 1 | 0.970 | 0.001 * |
| | 3 | 0.838 | 0.008 * |
| | 5 | 0.677 | 0.001 * |
| | 7 | 0.162 | <0.001 * |

* Denotes significant p-value (≤ 0.05). a 100 mg/kg/d CHIKV-EIG qd X 5, i.p., beginning 4 h before infection. b 50 mg/kg/d CHIKV-EIG qd X 5, i.p., beginning 4 h before infection. c 25 mg/kg/d CHIKV-EIG qd X 5, i.p., beginning 4 h before infection. d 100 mg/kg/d CHIKV-EIG qd X 3, i.p., beginning 4 h before infection.

**Table S5.** Summary statistics of cytokine levels at 6 dpi in right hind limb tissue cytokine levels in immunocompetent DBA/1J mice and comparison between CHIKV-EIG treatment groups and vehicle and methotrexate controls.

| Cytokine | Treatment Group | Mean (SD) | Median (25th, 75th percentile) | Min, Max | Wilcoxon Rank-Sum Test vs Vehicle | Wilcoxon Rank-Sum Test Vs Methotrexate |
|---|---|---|---|---|---|---|
| GMCSF | 1 [a] | 20.717 (54.811) | 0.00 (0.00, 0.00) | 0.00, 145.02 | 0.391 | 1.000 |
| | 3 [b] | 0.000 (0.000) | 0.00 (0.00, 0.00) | 0.00, 0.00 | 1.000 | 0.391 |
| | 5 [c] | 0.000 (0.000) | 0.00 (0.00, 0.00) | 0.00, 0.00 | 1.000 | 0.391 |

| | | | | | | |
|---|---|---|---|---|---|---|
| | 7 [d] | 0.000 (0.000) | 0.00 (0.00, 0.00) | 0.00, 0.00 | 1.000 | 0.391 |
| | 9 [e] | 0.000 (0.000) | 0.00 (0.00, 0.00) | 0.00, 0.00 | -- | -- |
| | 11[f] | 0.774 (2.048) | 0.00 (0.00, 0.00) | 0.00, 5.42 | -- | -- |
| IFNγ | 1 | 125.937 (40.193) | 128.23 (87.16, 150.88) | 68.76, 190.96 | 0.523 | 0.523 |
| | 3 | 123.775 (20.436) | 125.04 (117.96, 142.29) | 82.87, 143.45 | 0.250 | 0.371 |
| | 5 | 119.751 (29.265) | 131.01 (91.15, 136.21) | 74.43, 159.72 | 0.798 | 0.523 |
| | 7 | 75.814 (6.689) | 77.19 (72.67, 78.64) | 63.15, 84.84 | 0.002* | 0.018* |
| | 9 | 117.522 (16.300) | 115.63 (105.63, 122.59) | 95.24, 147.30 | -- | -- |
| | 11 | 110.480 (24.099) | 109.70 (83.32, 132.12) | 75.78, 137.68 | -- | -- |
| IL10 | 1 | 102.453 (133.102) | 17.10 (0.00, 188.36) | 0.00, 336.72 | 0.229 | 0.642 |
| | 3 | 77.480 (101.994) | 0.00 (0.00, 153.97) | 0.00, 245.72 | 0.372 | 1.000 |
| | 5 | 99.388 (122.262) | 76.08 (0.00, 186.35) | 0.00, 334.37 | 0.133 | 0.513 |
| | 7 | 22.622 (59.852) | 0.00 (0.00, 0.00) | 0.00, 158.35 | 0.722 | 0.180 |
| | 9 | 23.040 (39.556) | 0.00 (0.00, 73.63) | 0.00, 87.65 | -- | -- |
| | 11 | 45.418 (61.555) | 27.84 (0.00, 81.60) | 0.00, 167.52 | -- | -- |
| IL12p70 | 1 | 169.855 (45.912) | 187.92 (127.64, 193.58) | 86.60, 219.16 | 0.250 | 0.798 |
| | 3 | 148.145 (24.442) | 151.95 (147.49, 160.39) | 96.83, 175.43 | 0.482 | 0.250 |
| | 5 | 156.580 (34.342) | 171.03 (128.89, 181.65) | 91.64, 184.41 | 0.307 | 1.000 |
| | 7 | 99.459 (17.726) | 97.37 (91.64, 102.70) | 77.76, 135.49 | 0.030* | 0.030* |
| | 9 | 143.567 (34.775) | 149.09 (126.20, 158.26) | 81.46, 196.44 | -- | -- |
| | 11 | 168.940 (55.993) | 166.05 (114.90, 226.91) | 88.97, 246.32 | -- | -- |
| IL17 | 1 | 18.568 (45.699) | 0.00 (0.00, 7.99) | 0.00, 121.98 | 0.936 | 0.594 |
| | 3 | 0.000 (0.000) | 0.00 (0.00, 0.00) | 0.00, 0.00 | 0.173 | 0.391 |
| | 5 | 0.000 (0.000) | 0.00 (0.00, 0.00) | 0.00, 0.00 | 0.173 | 0.391 |
| | 7 | 0.000 (0.000) | 0.00 (0.00, 0.00) | 0.00, 0.00 | 0.173 | 0.391 |
| | 9 | 3.656 (9.414) | 0.00 (0.00, 0.59) | 0.00, 25.00 | -- | -- |
| | 11 | 2.142 (5.666) | 0.00 (0.00, 0.00) | 0.00, 14.99 | -- | -- |
| IL1a | 1 | 3920.307 (1903.751) | 4165.18 (2868.63, 5284.86) | 529.98, 6250.17 | 0.523 | 0.898 |
| | 3 | 5521.166 (1690.683) | 5792.18 (4399.35, 7121.65) | 2895.17, 7750.56 | 0.055 | 0.160 |
| | 5 | 3715.351 (1197.770) | 3015.20 (2908.35, 4451.52) | 2572.84, 5918.00 | 0.609 | 0.898 |
| | 7 | 2548.967 (950.348) | 2380.09 (1872.76, 2745.72) | 1767.11, 4556.69 | 0.125 | 0.25 |
| | 9 | 3773.296 (1884.169) | 3467.39 (2455.56, 3996.84) | 2147.15, 7718.30 | -- | -- |
| | 11 | 3877.762 (1808.493) | 4836.24 (1649.27, 5269.20) | 1576.16, 5939.99 | -- | -- |
| IL1b | 1 | 157.959 (40.677) | 160.92 (134.75, 194.64) | 85.52, 202.61 | 0.798 | 0.798 |
| | 3 | 163.063 (34.780) | 173.20 (142.44, 187.74) | 94.96, 195.10 | 0.443 | 0.701 |
| | 5 | 195.598 (61.430) | 202.33 (142.44, 255.27) | 95.87, 256.47 | 0.125 | 0.160 |
| | 7 | 98.453 (19.975) | 91.51 (83.88, 109.20) | 78.29, 137.67 | 0.015* | 0.084 |
| | 9 | 152.341 (34.745) | 159.57 (130.36, 184.73) | 93.71, 194.92 | -- | -- |
| | 11 | 145.542 (50.605) | 167.03 (91.51, 190.32) | 78.64, 203.76 | -- | -- |
| IL2 | 1 | 6.656 (12.414) | 0.00 (0.00, 14.65) | 0.00, 31.94 | 0.722 | 0.722 |
| | 3 | 14.931 (23.047) | 0.00 (0.00, 24.38) | 0.00, 61.77 | 0.337 | 0.424 |
| | 5 | 2.887 (7.639) | 0.00 (0.00, 0.00) | 0.00, 20.21 | 1.000 | 1.000 |
| | 7 | 0.000 (0.000) | 0.00 (0.00, 0.00) | 0.00, 0.00 | 0.391 | 0.391 |
| | 9 | 7.300 (19.315) | 0.00 (0.00, 0.00) | 0.00, 51.10 | -- | -- |
| | 11 | 13.172 (34.849) | 0.00 (0.00, 0.00) | 0.00, 92.20 | -- | -- |

| | | | | | | |
|---|---|---|---|---|---|---|
| IL3 | 1 | 67.514 (18.788) | 66.47 (56.29, 81.17) | 33.87, 89.92 | 0.898 | 0.250 |
| | 3 | 68.173 (13.759) | 72.76 (64.35, 77.76) | 39.62, 78.45 | 0.701 | 0.160 |
| | 5 | 65.490 (24.156) | 63.13 (41.68, 72.31) | 38.18, 111.27 | 1.000 | 0.482 |
| | 7 | 41.479 (4.208) | 41.18 (39.62, 44.22) | 35.00, 48.68 | 0.030 * | 0.523 |
| | 9 | 67.407 (21.927) | 65.92 (54.52, 81.96) | 33.33, 103.20 | -- | -- |
| | 11 | 54.798 (19.205) | 52.76 (37.72, 76.03) | 32.81, 77.60 | -- | -- |
| IL4 | 1 | 61.200 (18.187) | 62.70 (51.91, 79.00) | 27.35, 81.80 | 0.097 | 0.055 |
| | 3 | 48.214 (7.590) | 49.87 (47.99, 52.99) | 32.00, 54.88 | 0.798 | 0.898 |
| | 5 | 54.154 (17.839) | 52.79 (43.85, 55.41) | 30.84, 89.55 | 0.443 | 0.749 |
| | 7 | 30.756 (1.680) | 30.84 (29.24, 32.00) | 28.27, 33.25 | 0.030 * | 0.084 |
| | 9 | 49.105 (14.004) | 48.96 (42.80, 54.62) | 26.92, 73.69 | -- | -- |
| | 11 | 45.764 (14.253) | 47.93 (30.84, 60.16) | 26.50, 60.17 | -- | -- |
| IL5 | 1 | 142.396 (36.042) | 153.11 (98.32, 172.96) | 88.81, 177.39 | 1.000 | 0.523 |
| | 3 | 157.817 (24.655) | 164.06 (155.82, 172.05) | 103.89, 174.88 | 0.443 | 0.097 |
| | 5 | 160.181 (49.235) | 167.00 (100.11, 197.19) | 98.32, 227.86 | 0.898 | 0.337 |
| | 7 | 107.396 (27.942) | 100.11 (91.77, 107.96) | 83.42, 168.23 | 0.073 | 0.482 |
| | 9 | 158.177 (44.612) | 152.94 (135.39, 211.73) | 87.40, 216.71 | -- | -- |
| | 11 | 143.066 (71.313) | 126.50 (86.03, 161.93) | 84.71, 288.93 | -- | -- |
| IL6 | 1 | 69.056 (17.820) | 73.38 (55.25, 77.88) | 41.03, 96.62 | 0.522 | 0.798 |
| | 3 | 71.961 (11.116) | 74.82 (72.00, 79.50) | 48.00, 81.08 | 0.084 | 0.481 |
| | 5 | 84.762 (23.765) | 83.62 (72.00, 94.95) | 46.25, 125.04 | 0.041 * | 0.201 |
| | 7 | 59.162 (29.948) | 48.00 (42.40, 61.09) | 38.55, 125.04 | 0.125 | 0.030 * |
| | 9 | 66.108 (12.038) | 70.67 (62.56, 74.28) | 40.38, 74.82 | -- | -- |
| | 11 | 74.243 (12.529) | 71.67 (66.36, 76.00) | 62.23, 100.48 | -- | -- |
| MCP1 | 1 | 42.941 (14.645) | 41.93 (34.24, 46.36) | 22.10, 70.16 | 0.003 * | 0.002 * |
| | 3 | 124.345 (185.652) | 52.91 (41.16, 73.43) | 40.28, 544.33 | 0.055 | 0.030 * |
| | 5 | 47.151 (23.062) | 39.04 (36.82, 47.56) | 24.46, 96.48 | 0.005 * | 0.002 * |
| | 7 | 52.455 (61.163) | 26.86 (25.85, 45.70) | 24.46, 190.17 | 0.021 * | 0.005 * |
| | 9 | 151.587 (75.680) | 154.49 (91.17, 169.41) | 54.90, 295.74 | -- | -- |
| | 11 | 286.985 (124.803) | 286.91 (187.82, 440.06) | 129.56, 447.13 | -- | -- |
| MIP1a | 1 | 92.162 (243.837) | 0.00 (0.00, 0.00) | 0.00, 645.13 | 1.000 | 1.000 |
| | 3 | 11.278 (22.547) | 0.00 (0.00, 19.17) | 0.00, 59.77 | 0.594 | 0.477 |
| | 5 | 17.817 (26.403) | 0.00 (0.00, 36.93) | 0.00, 68.26 | 0.200 | 0.200 |
| | 7 | 0.000 (0.000) | 0.00 (0.00, 0.00) | 0.00, 0.00 | 0.391 | 0.391 |
| | 9 | 2.741 (7.251) | 0.00 (0.00, 0.00) | 0.00, 19.19 | -- | -- |
| | 11 | 2.692 (7.122) | 0.00 (0.00, 0.00) | 0.00, 18.84 | -- | -- |
| RANTES | 1 | 26.707 (62.312) | 0.00 (0.00, 19.94) | 0.00, 167.01 | 0.019 * | 0.006 * |
| | 3 | 34.377 (42.678) | 17.58 (0.00, 88.76) | 0.00, 98.74 | 0.040 * | 0.005 * |
| | 5 | 59.635 (23.654) | 59.09 (40.28, 88.39) | 30.68, 89.54 | 0.250 | 0.007 * |
| | 7 | 726.037 (1848.792) | 23.57 (0.00, 83.96) | 0.00, 4918.20 | 0.125 | 0.041 * |
| | 9 | 156.005 (162.435) | 90.68 (46.79, 311.21) | 19.03, 451.27 | -- | -- |
| | 11 | 160.056 (56.369) | 176.47 (130.71, 193.49) | 62.38, 240.15 | -- | -- |
| TNF$\alpha$ | 1 | 54.051 (28.722) | 49.98 (41.65, 68.34) | 11.97, 106.59 | 0.609 | 0.201 |
| | 3 | 39.885 (17.517) | 30.44 (26.04, 53.45) | 22.58, 69.90 | 0.609 | 0.523 |
| | 5 | 57.227 (18.711) | 51.87 (43.36, 76.53) | 29.96, 80.30 | 0.307 | 0.125 |
| | 7 | 138.426 (265.366) | 33.66 (13.25, 90.61) | 12.37, 736.87 | 0.798 | 0.898 |
| | 9 | 53.565 (36.467) | 46.68 (31.33, 51.28) | 22.46, 132.94 | -- | -- |

| | | | | | |
|---|---|---|---|---|---|
| 11 | 39.505 (29.853) | 30.86 (17.48, 45.81) | 16.53, 101.38 | -- | -- |

* Denotes significant p-value (≤ 0.05). p-values are not adjusted for multiple comparisons. a 100 mg/kg/d CHIKV-EIG qd X 5, i.p., beginning 4 h before infection. b 50 mg/kg/d CHIKV-EIG qd X 5, i.p., beginning 4 h before infection. c 25 mg/kg/d CHIKV-EIG qd X 5, i.p., beginning 4 h before infection. d 100 mg/kg/d CHIKV-EIG qd X 3, i.p., beginning 4 h before infection. e 2 mg/kg/d methotrexate qd X 5, s.c., beginning 1 day before infection. f 100 μL PBS (vehicle) qd X 5, i.p., beginning 4 h before infection.
